# Detection of nitro-aromatics using C_5_N_2_ as an electrochemical sensor: a DFT approach†

**DOI:** 10.1039/d4ra05600k

**Published:** 2024-09-23

**Authors:** Nabeela, Muhammad Ali Hashmi, Ahmad Nauman Shah Saqib, Aqsa Kamran, Ahmed Lakhani

**Affiliations:** a Department of Chemistry, Division of Science & Technology, University of Education Lahore 54770 Pakistan muhammad.hashmi@ue.edu.pk; b School of Chemical and Physical Sciences, Victoria University of Wellington Wellington 6012 New Zealand; c Department of Biomedical and Health Sciences, Calumet College of St. Joseph 2400, New York Ave Whiting IN 46394 USA alakhani@ccsj.edu

## Abstract

Nitroaromatics impose severe health problems and threats to the environment. Therefore, the detection of such hazardous substances is essential to save the whole ecosystem. Herein, the C_5_N_2_ sheet is used as an electrochemical sensor for the detection of 1,3-dinitrobenzene (1,3-DNB), trinitrotoluene (TNT), and picric acid (PA) using the PBE0/def2SVP level of theory as implemented in Gaussian 16. The highest interaction energy was observed for the picric acid@C_5_N_2_ complex. The trend in interaction energies for the studied system is PA@C_5_N_2_ >TNT@C_5_N_2_ >1,3-DNB@C_5_N_2_. The studied systems were further analysed by qualitative and quantitative analyses to determine the interactions between the nitroaromatic analytes and the C_5_N_2_ sheet. Electronic properties of all analytes@C_5_N_2_ complexes have been examined by NBO, EDD, FMO and DOS analysis. QTAIM analysis depicts the stronger non-covalent interactions for the PA@C_5_N_2,_ which shows consistency with interaction energy and NCI analysis. Furthermore, NBO and FMO analyses show that the C_5_N_2_ substrate exhibits high sensitivity and selectivity towards the picric acid compared to TNT and 1,3-DNB nitroaromatics. EDD and DOS analyses are in agreement with NBO and FMO analyses. Furthermore, the recovery time of the studied system has been computed to determine the efficiency of C_5_N_2_ material as an electrochemical sensor. Overall, the results show that carbon nitride can be a good sensor for the detection of nitroaromatics.

## Abstract 2

Nitroaromatics are hazardous pollutants. This study investigates C_5_N_2_ as a sensor for 1,3-DNB, TNT, and picric acid using DFT. Picric acid exhibited the strongest interaction, indicating C_5_N_2_'s potential as a selective sensor for nitroaromatics.

## Graphical abstract

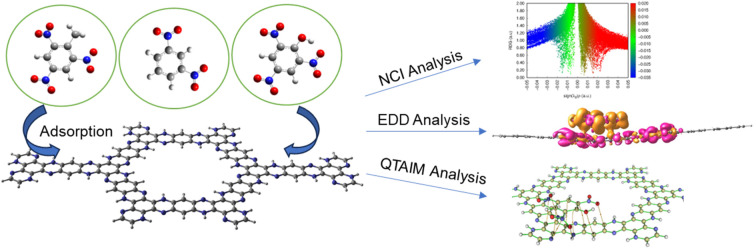

## 1 Introduction

In recent years, extensive use of explosives in defence systems has raised serious concerns as they have potential negative impacts on human health and the whole ecosystem.^1^ Among these, nitroaromatics such as 1,3-dinitrobenzene (DNB), 2,4,6-trinitrophenol, also known as picric acid (PA), and trinitrotoluene (TNT), have been widely used due to their highly explosive nature. Besides, these nitroaromatics have also gained popularity in the synthetic industry, including pharmaceuticals, pigments, dyes, pesticides, wood preservatives, and rubber chemicals.^2–5^ Release of these compounds, intentionally or unintentionally, contaminates the environment and deteriorates human health by causing problems such as skin irritation, cataracts, cancer, anaemia, cyanosis, and male infertility, damaging the liver, kidney, and blood cells in humans.^6–8^ Therefore, detection of such toxic compounds is crucial to overcome their potential threats.

Various techniques such as HPLC, gas chromatography,^9^ ion mobility spectrometry,^10–12^ and capillary electrophoresis^13,14^ have already been used for detection purposes. However, their non-specificity, difficulty in handling the instruments, pre-sample processes, expensive instrumentations, and portability hinder their applicability as detectors.^15,16^ The demand for rapid, responsive, portable, and flexible detecting systems marked the electrochemical sensors as a potential alternative for efficient detection.^17^

Porous materials have gained significant importance as electrochemical sensors due to their exceptional sensing properties^18^ and various 2D and 3D substrates have been evaluated both experimentally and theoretically for this purpose. Such substances include fullerene,^19^ metal–organic frameworks (MOFs),^20^ aluminium nitride,^21^ silicon carbide,^22^ ZnO nanotubes,^23^ silver–graphene quantum dots,^24^ metal-doped graphene,^25,26^ and covalent organic frameworks.^27^ In addition to these, graphene–polyaniline,^28^ polyaniline/ZnO,^29^ single-walled nanotubes,^30^ multi-walled nanotubes,^31^ polypyrrole,^32^ and h-BN nanoclusters^33^ have been used for detecting the chemical warfare agents (CWAs) including nitro explosives. Although these adsorbents are used to sense toxic agents, they lack certain characteristics, such as large surface area coupled with more active sites for adsorption, high porosity and reproducible results, required for the efficient performance of the adsorbents.^34^

Recently, carbon nitride became the heart of sensors due to its specific surface area and highly porous and chemically stable structure. Moreover, the intrinsic bandgap of carbon nitride proves its efficacy as a catalyst in water-splitting reactions, carbon dioxide reduction reactions, and degradation of organic pollutants.^35,36^ Numerous experimental and theoretical studies have been performed on the sensing capability of carbon nitride having different ratios of carbon and nitrogen, such as C_2_N, C_3_N_4,_ and C_4_N nanoflakes.^37^ These materials show excellent sensing performance in the detection of toxic agents containing ammonia, hydrogen sulphides,^38^ pesticides,^39^ nitrogen halides,^40^ neurotoxin hydrazine derivatives,^41^ CWAs,^42,43^ and nitro-explosives.^44,45^

An innovative form of carbon nitride, *i.e.* C_5_N_2,_ has not yet been explored for sensing. The C_5_N_2_ material is synthesised through a condensation reaction between 1,2,4,5-benzene tetramine and hexaketocyclohexane.^46,47^ Theoretical studies showed that C_5_N_2_ is used as a catalyst to determine its stability and activity for the synthesis of hydrogen peroxide (H_2_O_2_).^48^ Moreover, C_5_N_2_ is superior to the other carbon nitride forms due to its nanoporous crystalline structure with a narrow band gap (1.10 eV).^49^ These findings motivated us to explore the sensing capability of the C_5_N_2_ toward the nitroaromatic compounds, *i.e.*, PA, 1,3-DNB and TNT. DFT calculations have been performed to determine the adsorption of the analytes on the C_5_N_2_ surface. The selectivity and sensitivity of the analytes on the C_5_N_2_ surface have been investigated by the FMO, NBO and DOS analyses. Furthermore, the nature of interactions is explored through the QTAIM and NCI analyses.

## 2 Computational methodology

Quantum chemistry computational calculations have been performed using Gaussian 16 B.01.^50^ Structures of analystes, C_5_N_2_ and complexes have been modelled using Gauss View software.^51^ Geometries of the substrate C_5_N_2_ and nitro-containing analytes were optimised at the PBE0-D3BJ/def2-SVP level of theory. PBE0 is a hybrid DFT functional, which outperforms the pure DFT approach in computing molecular structures and properties throughout the periodic table.^52^ The literature has also backed the selection of PBE0 functional as it is widely applicable for molecular systems, providing accurate results for atomic energies, structural geometry, dissociation energies, and electronic and magnetic properties^53^ and sensing interactions.^54^ PBE0/def2XVP has been used for adsorption studies of CO_2_,^55^ metformin,^56^ and different gases including H_2_, N_2_, CO, H_2_S, NH_3_, SO_2_, and CH_4_ ^57^ over graphitic carbon nitride and to study the non-covalent interactions between the decavanadate, arginine, and lysine side chains in proteins.^58^ Similarly, PBE0 has also been studied with different basis set combinations to study Se-doped graphitic carbon nitride (Se@gC_3_N_4_) nanostructures used for the smart therapeutic delivery of zidovudine,^59^ for the efficient sensing of glucose towards borophene,^53^ functionalized carbon quantum dots for sensing curcumin,^60^ and in studying the sensing of telomerase through semiconducting carbon nitrides.^61^ In a study about adsorption behaviour for efficient sensing of melamine through Mg_12_O_11_–X nanostructured materials, different hybrid functionals have been employed, and PBE0 has been reported to be ranked at the top in describing the sensing interactions.^54^ Considering the long-range non-covalent interactions, Grimme's empirical D3 correction^62^ with Becke–Johnston damping (D3BJ)^63^ has been applied in all calculations. Multiwfn (version 3.80)^64^ and VMD software^65^ have been used for qualitative and quantitative analyses. CYLview^66^ has been employed to obtain high-resolution visualisation of modelled structures.

Analytes with different orientations on the C_5_N_2_ sheet have been optimised to obtain the lowest energy structure of the complexes. These optimised structures are further validated *via* the vibrational frequency analysis, ensuring they have no imaginary frequency. The interaction energies of analytes with the sheet have been calculated through the following formula:

1 Δ*E*_int_ = [*E*_analyte@C_5_N_2__ − (*E*_analyte_ + *E*_C_5_N_2__)]

Δ*E*_int_ represents the interaction energy of the analyte@C_5_N_2_ substrate, whereas the energy of complex, analyte, and C_5_N_2_ sheet, respectively, are shown by the terms *E*_analyte@C_5_N_2__, *E*_analyte_, and *E*_C_5_N_2__.

The interactions between the C_5_N_2_ substrate and the nitro analytes have been thoroughly analyzed using Non-Covalent Interactions (NCI) analysis. This method primarily utilizes electron density (*ρ*) and the Reduced Density Gradient (RDG) to probe these interactions. The RDG is calculated using the following equation:^67^2
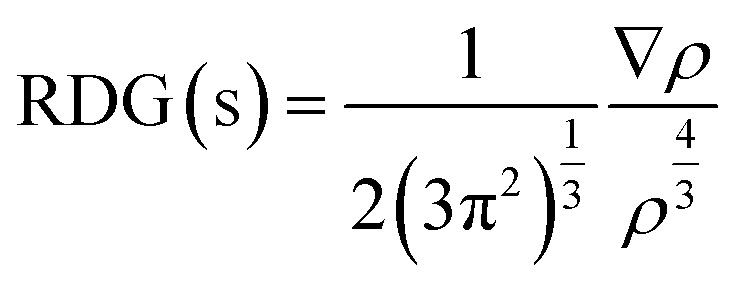


This equation indicates that the RDG value is inversely proportional to the electron density at a point. A higher electron density generally leads to a lower RDG value, suggesting a stronger potential for non-covalent bonding at that location.

Additionally, the electron density magnitude influences these non-covalent interactions' strength. In contrast, the interaction's specific nature—whether repulsive or attractive—is determined by the Laplacian of the electron density (∇^2^*ρ*). The Laplacian is expressed as the sum of its three eigenvalues from the Hessian matrix of electron density:^24^

3 ∇^2^*ρ* = *λ*_1_ + *λ*_2_ + *λ*_3_

Among the eigenvalues derived from the Hessian matrix, the second eigenvalue (*λ*_2_) is especially important for determining the nature of the interactions within the molecule. Specifically, a negative *λ*_2_ indicates strong, attractive interactions, such as those seen in hydrogen bonds, which are critical for molecular stability and function. Conversely, a positive *λ*_2_ points to repulsive interactions, which might indicate regions of molecular strain or steric clashes. Additionally, when *λ*_2_ is near zero, it typically signifies the presence of weaker interactions, like van der Waals forces, which are essential for subtler molecular phenomena.

To visually capture and illustrate these different types of non-covalent interactions, we employ 2D RDG graphs and 3D isosurfaces generated using Multiwfn Software (version 3.80).^64,68^ These visualizations are color-coded to enhance understanding and interpretation: red represents repulsive interactions, blue denotes strong attractive interactions (notably hydrogen bonds), and green highlights the van der Waals interactions. This color-coding aids in quickly identifying the nature of interactions at a glance, providing an intuitive map of molecular interaction landscapes.^69^

Furthermore, Quantum Theory of Atoms in Molecules (QTAIM) analysis has been performed to recognise the intramolecular and intermolecular interactions, particularly the non-covalent interactions.^70,71^ The nature and strength of intermolecular interactions between the nitroaromatic analytes and C_5_N_2_ substrate are determined by the various topological parameters such as electron density (*ρ*), kinetic and potential energy density (*G* and *V*), total electron energy density (*H*), and Laplacian of the electron density (∇^2^*ρ*), which exist at bond critical points (BCP) between nitroaromatic analytes and the substrate.^69^

4 *H*(*r*) = *V*(*r*) + *G*(*r*)

Generally, the values of potential energy density and kinetic energy density are always negative and positive, respectively. The sum of these two parameters (*V* and *G*) gives the total energy density (*H*). The values of *H* and ∇^2^*ρ* depict the type of interactions either covalent or non-covalent. If the value is *H* > 0 < ∇^2^*ρ*, then it shows the non-covalent interactions, while covalent interactions are depicted by the *H* < 0 > ∇^2^*ρ*.^71,72^ Electronic density values further specify the strength of non-covalent interactions. If the value of electronic density is greater than 0.1, then it indicates the electrostatic attractive forces, while the presence of van der Waals interactions is indicated by the value of electronic density lower than 0.1.^73^

In addition, the electronic properties of the analytes@C_5_N_2_ complexes have been determined at the same level of theory through frontier molecular orbital (FMO), electron density distribution (EDD), density of states (DOS) and natural bond orbital (NBO) analysis. The density of states validates the FMO analysis by better understanding the energy gap after the adsorption of analytes. EDD plots of electronic density were obtained using Chemcraft.^74^ Moreover, NBO charges are confirmed by providing visual aids through EDD plots to understand charge transfer upon complexation.

## 3 Results and discussion

**Fig. 1:**
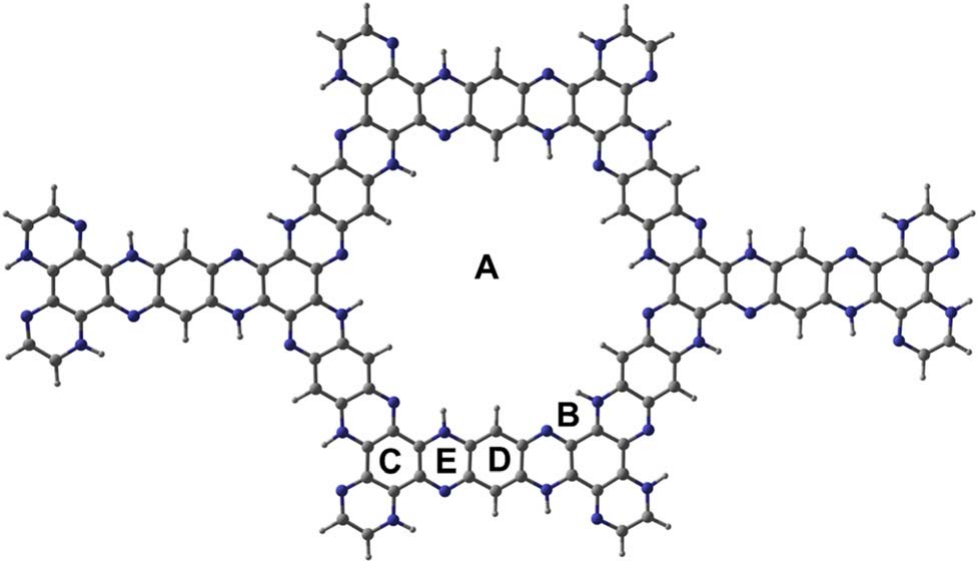
Optimised geometry of C_5_N_2_ at PBE0-D3BJ/def2SVP with five binding sites *i.e.*, central cavity (A), triazine site (B), benzene ring (C), hydrogenated benzene ring (D) and pyrazine ring (E). Blue color shows the nitrogen atom, grey shows the carbon atom and greyish-white shows the hydrogen.

Fig. 1 shows the optimised structure of C_5_N_2_ used as an adsorbent. C_5_N_2_ sheet consists of five fused benzene rings, bridged by a pyrazine ring, consisting of four different kind of bonds, depending on the type of atoms and ring involved. The C–N bond length in the pyrazine ring is 1.36 Å whereas the bond length of three types of C–C bonds is 1.4 Å, 1.42 Å and 1.38 Å present in pyrazine, benzene and hydrogenated benzene rings, respectively. The analytes (1,3-DNB, TNT and PA) were placed on five possible binding sites with different orientations to get the most stable ones. Such sites are labelled as A (in the central cavity of the sheet), B (forming a triangle with nitrogen atoms at the edges), C (top of the benzene ring), D (top of the pyrazine ring) and E (top of hydrogenated benzene ring). Analytes were positioned with possible orientations on the mentioned sites of the C_5_N_2_ sheet to get the most stable geometry of the complex.

**Fig. 2:**
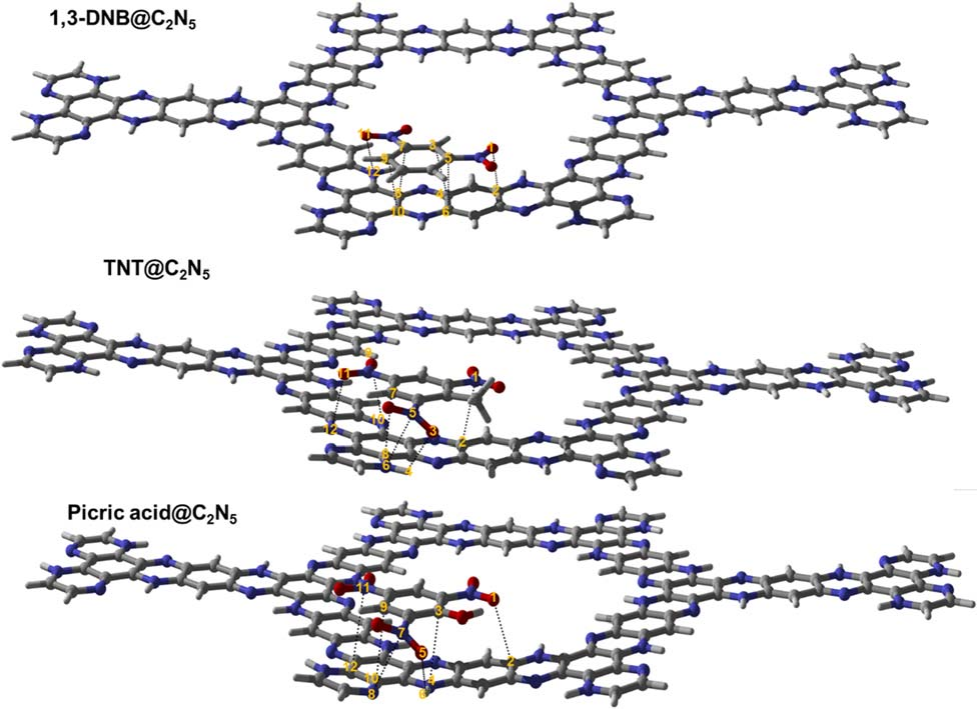
The stable optimised structure of 1,3-DNB@C_5_N_2_, TNT@C_5_N_2_ and picric acid@C_5_N_2_ at PBE0-D3BJ/def2SVP level of theory. Interacting atoms of analytes and substrate have been shown by dotted lines.

Among the possible orientations of the 1,3-DNB complex (see Fig. S1†), the most stable geometry occurred at the top of the pyrazine ring with an interaction energy of −23.21 kcal mole^−1^. The benzene ring of the 1,3-DNB analyte was located on the site D while the two nitro groups were situated on the site B and E of the C_5_N_2_ surface, respectively. For the TNT@C_5_N_2_ complex, the analyte was placed in five possible orientations on the C_5_N_2_ sheet (see Fig. S3†). Among these, the most stable geometry was obtained with an interaction energy of −31.64 kcal mole^−1^ where the analyte was oriented in between the C and D sites of the surface. The oxygen of TNT formed a hydrogen bond with the hydrogen pyrazine ring of C_5_N_2_ substrate (2.49 Å). The interaction energies of the possible orientation of analytes at C_5_N_2_ are also presented in Table S1.† Stable geometries of 1,3-DNB@C_5_N_2_, TNT@C_5_N_2_ and PA@C_5_N_2_ have been shown in Fig. 2.

Among all the three complex systems, picric acid interacts strongly with the surface of the substrate. For PA@C_5_N_2_ complexes, the most stable geometry is obtained when the analyte is located on the top of the D site (pyrazine ring) with −34.37 kcal mole^−1^ of interaction energy. The other unstable optimized structures are presented in Fig. S2.† One of the reasons for such a high interaction energy is the formation of a hydrogen bond between the oxygen of picric acid and hydrogen of the pyrazine ring. The interaction energies of TNT@C_5_N_2_ and PA@C_5_N_2_ are stronger than those of the 1,3-DNB@C_5_N_2_ complex due to the presence of hydrogen bond formation between the analyte and the substrate. Trends in interaction energies for the considered complexes are PA@C_5_N_2_ > TNT@C_5_N_2_ > 1,3-DNB@C_5_N_2_. The interaction energies along with bond lengths of all three complex systems have been presented in Table 1.

**Table 1:** Interaction energies of all the complexes in kcal mol^−1^ along with bond length (Å) of interacting atoms of analytes and C_5_N_2_ substrate

Analyte@C_5_N_2_ sheet complex	Interacting atoms of analytes and C_5_N_2_	Bond lengths (Å)	Interaction energies (kcal mol^−1^)
PA@C_5_N_2_	N7–N8	3.05	−34.37
	O5–H6	2.67	
	C3–N4	3.06	
	C9–C10	2.99	
	N11–C12	3.06	
	O1–C2	3.01	
TNT@C_5_N_2_	N5–N6	2.91	−31.64
	C7–C8	2.92	
	O11–N12	3.03	
	N1–C2	3.09	
	O9–N10	3.15	
	O3–H4	2.49	
1,3-DNB@C_5_N_2_	C7–C8	3.17	−23.21
	C9–C10	3.09	
	C5–C6	3.11	
	C3–C4	3.15	
	O1–C2	2.99	
	O11–N12	3.01	

### 3.1 Quantum theory of atoms in molecules analysis

**Fig. 3:**
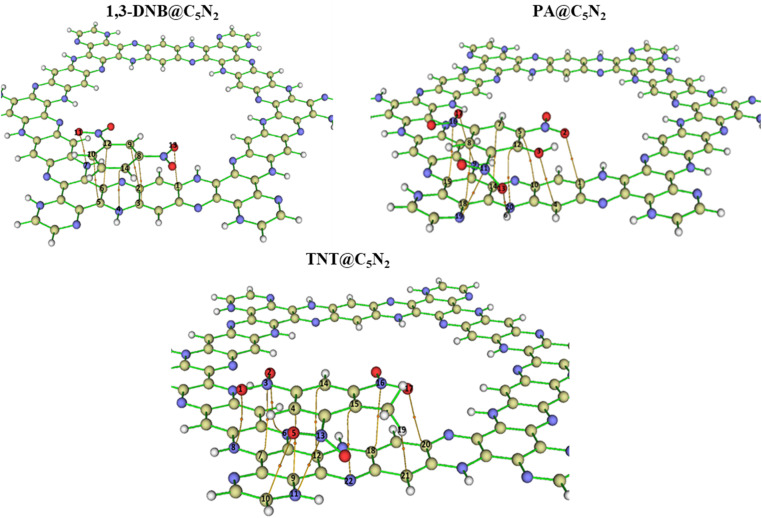
Visual representation of BCPs *via* QTAIM analysis of the considered analytes@C_5_N_2_ complexes.

The nature of intermolecular interactions in nitroaromatics@C_5_N_2_ complexes is analysed through the quantum theory of atoms in molecules (QTAIM) analysis. For the 1,3-DNB@C_5_N_2_ complex, seven BCPs with four C–C, one C–N, one C–O, and one N–O were identified between the 1,3-DNB and C_5_N_2_ substrate, as shown in Fig. 3. The value of Laplacian of electron density ranges from 0.019 to 0.033, signifying the non-covalent interactions, while electron density exhibits a value less than 0.1 (0.008 to 0.011), depicting the van der Waals interactions between the 1,3-DNB and C_5_N_2_ sheet. Additionally, the remaining topological parameter values, *i.e.*, H, G and V of 1,3-DNB@C_5_N_2_ also indicate the presence of non-covalent interactions (Table S2†). For the TNT@C_5_N_2_, eleven BCPs with two C–C, three C–N, two C–O, one C–H, one N–N, and two N–O were observed, as shown in Fig. 3. Topological parameters at the mentioned BCPs show non-covalent interactions between the TNT and C_5_N_2_ substrate. The values of Laplacian and electron density range from 0.023 to 0.037 and 0.007 to 0.014, respectively. The remaining topological parameters, *i.e.*, H, G and V of TNT@C_5_N_2_ also indicated the presence of non-covalent interactions (see Table S1†).

In the case of PA@C_5_N_2,_ ten BCPs are obtained with one O–H, two C–O, three C–C, two C–N, one N–N, and one N–O, between the picric acid and substrate (see Fig. 3). The topological parameters of PA@C_5_N_2_ were similar to the TNT@C_5_N_2_ except for the one O–H BCP. For all the BCPs, values of Laplacian and electron density range from 0.023 to 0.033 and 0.005 to 0.012, respectively. These topological values were in good agreement with the non-covalent interactions, particularly the van der Waals interactions. The value of electron density was highest for BCPs between the C8 of the picric acid and C18 of the C_5_N_2_ surface which depicted the strong non-covalent interactions. The rest of topological parameters (H, G, V) also showed consistency with values of Laplacian and electron density as reported in Table 2.

**Table 2:** QTAIM topological parameters for PA@C_5_N_2_. Topological parameters are *ρ* (electron density), ∇2*ρ* (Laplacian of electron density), *G* (kinetic energy density), *V* (potential energy density) and *H* (total electron energy density)

Analyte–C_5_N_2_	∇_2_*ρ*	*ρ*	*V*(*r*)	*H*	*G*(*r*)
PA@C_5_N_2_					
N11–N19	0.027	0.0082	−0.005	0.001	0.006
C8–C18	0.033	0.0121	−0.006	0.001	0.007
O2–C1	0.031	0.0101	−0.006	0.001	0.007
O13–H6	0.023	0.0055	−0.004	0.001	0.005
O17–N9	0.024	0.0069	−0.005	0.001	0.005
N16–C15	0.030	0.0085	−0.005	0.001	0.006
C12–N20	0.028	0.0099	−0.005	0.001	0.006
O3–C4	0.025	0.0084	−0.005	0.001	0.005
C7–C14	0.026	0.0091	−0.004	0.001	0.005
C5–C10	0.027	0.0096	−0.005	0.001	0.006

Topological parameters for all considered complexes indicated the presence of non-covalent interaction between the analytes and the C_5_N_2_ substrate. Values of electron density and Laplacian for PA@C_5_N_2_ complexes showed van der Waals interactions along with hydrogen bond interactions. These QTAIM results are consistent with the interaction energies.

### 3.2 Non-covalent interaction analysis

The intermolecular interactions have been further characterised by the NCI analysis in the complexes of analytes–C_5_N_2_. It comprises of 2D RDG graph and 3D isosurfaces. Three color schemes have been used in 3D isosurface which describes the non-covalent interactions *i.e.*, blue, green and red for attractive, weak van der Waals, and dispersive interactions between the analytes and substrate. Similarly, the 2D RDG graph shows attractive interactions in the form of hydrogen bonding (blue spikes), repulsive force (red spikes) and weak van der Waals forces (green spikes). Whereas the size of a particular patch is directly related to the strength of interactions. The larger the size of a particular patch, the greater will be the strength of that interaction. Similarly, values of electron density on the *x*-axis vary directly with the nature of interactions between the analytes and the substrate.

**Fig. 4:**
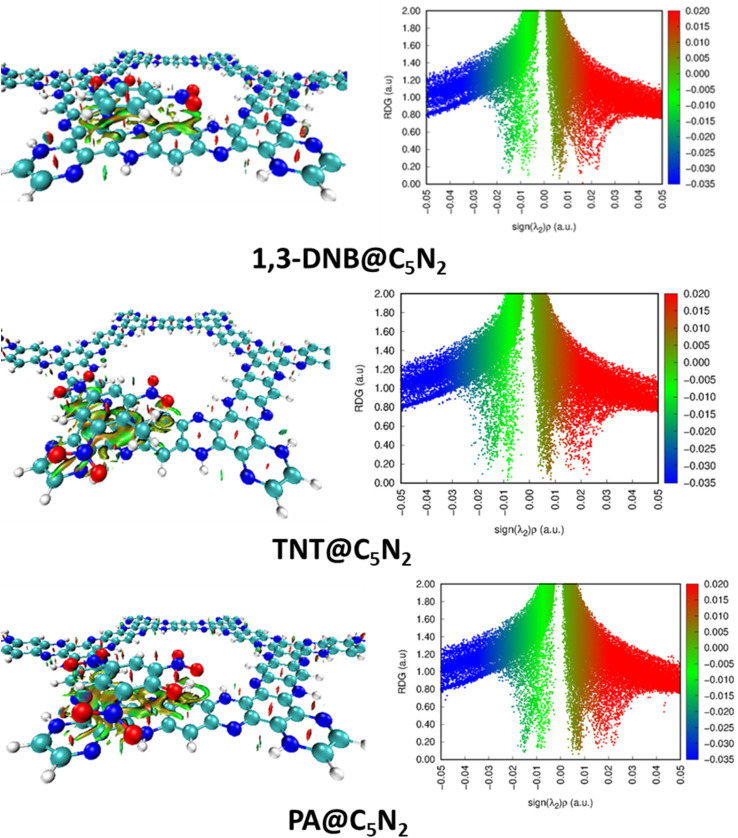
3D isosurface and 2D RDG graph of nitroaromatics analytes@C_5_N_2_ complexes.

In the 3D isosurface, the presence of green isosurface between the analytes (1,3-DNB, TNT and PA) and the C_5_N_2_ substrate confirms the weak van der Waals interactions consistent with the 2D RDG graph. Conversely, there is no hydrogen bond interaction between the analytes and substrate as revealed by the absence of clear blue patches.^75^ However, dispersed bluish-green spots in the case of TNT@C_5_N_2_ and PA@C_5_N_2_ showed more van der Waals interactions. 2D RDG and 3D isosurface are presented in Fig. 4. Moreover, the presence of red spikes (0.01 to 0.03) in the 2D RDG graph represents the repulsive interaction that mostly exists between the atoms of aromatic rings of the C_5_N_2_ substrate, also depicted in the 3D isosurface.^76^ In the case of picric acid@C_5_N_2_ complex, the green isosurface and spikes in 3D and 2D graphs respectively, are dense and wide as compared to 1,3-DNB and TNT complexes, reflecting more van der Waals interactions between the picric acid and C_5_N_2_ which is also consistent with the interaction energies and QTAIM topological results.

### 3.3 Electronic properties: FMO and DOS analysis

**Fig. 5:**
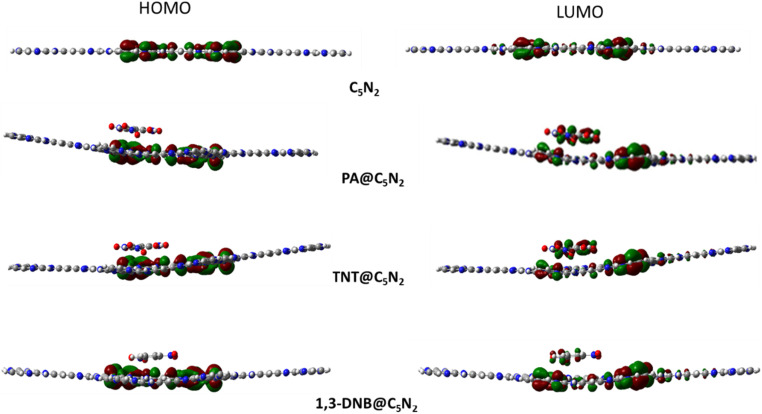
HOMO–LUMO orbitals densities of the pristine C_5_N_2_, PA@C_5_N_2_, TNT @C_5_N_2_ and 1,3-DNB@C_5_N_2_.

Electronic properties of the substrate upon complexation have been studied through FMO analysis. After the analytes' adsorption, the substrate's conductivity significantly changed, which influenced its sensing capability. FMO analysis for the considered analytes@C_5_N_2_ complexes has been performed. The energy values of HOMO, LUMO and their energy gaps have been reported in Table 3. Orbital density isosurface of all the analytes@complexes are shown in Fig. 5. Analysis shows that HOMO and LUMO values for pristine C_5_N_2_ substrate are −3.42 eV and −2.82 eV respectively, which results in an energy gap of 0.60 eV. The energy gap of the pristine C_5_N_2_ has been changed after complexation with the analytes (1,3-DNB, TNT and PA). HOMO and LUMO energy values of 1,3-DNB@C_5_N_2_ have been increased to −3.41 eV and −2.94 eV, respectively which results in decreased value of *E*_H–L_ gap (0.47 eV). After adsorption of TNT analytes upon C_5_N_2_, the HOMO–LUMO energy gap reduces to 0.46 eV similar to the 1,3-DNB@C_5_N_2_ complex. In this case, the decrease in *E*_H–L_ gap is now attributed to the decreased value of both HOMO (−3.47 eV) and LUMO (−3.01 eV) as compared to the pristine C_5_N_2_ substrate. Among all the studied systems, a significant reduction in the HOMO–LUMO energy gap is observed in the case of PA@C_5_N_2_. The value of HOMO is reduced to −3.47 eV, comparable to the HOMO value of the pristine C_5_N_2_. However, pronounced shift in E_H–L_ is mainly due to the reduction in the value of LUMO (−3.10 eV). This noticeable reduction in energy gap (0.37 eV) results in excellent conductivity and sensitivity of C_5_N_2_ substrate towards the picric acid as compared to the other analytes (1,3-DNB and TNT).^77^

**Table 3:** HOMO–LUMO energies and their energy gap (eV) of all the three analytes–complexes and bare C_5_N_2_ sheet along with NBO results

Analyte@C_5_N_2_	Pristine C_5_N_2_	PA@C_5_N_2_	TNT@C_5_N_2_	1,3-DNB @C_5_N_2_
HOMO (eV)	−3.42	−3.47	−3.47	−3.41
LUMO (eV)	−2.82	−3.10	−3.01	−2.94
*E*_H–L_ gap (eV)	0.60	0.37	0.46	0.47
NBO charge (*e*^−^)		−0.426	−0.406	−0.228

Orbital density patterns for considered analytes@C_5_N_2_ complexes obtained through FMO analysis are shown in Fig. 5. Orbital density distribution shows similar patterns in all three analytes@C_5_N_2_ complex systems. For all three systems (1,3-DNB@C_5_N_2_, TNT@C_5_N_2_, PA@C_5_N_2_), the HOMO isosurface lies entirely on the sheet depicting the transfer of charge density from the C_5_N_2_ surface after adsorption of analytes. The LUMO density orbital is found half on the analyte and half on the substrate which shows a significant reduction in the HOMO–LUMO energy gap. Among the 1,3-DNB@C_5_N_2_ and TNT@C_5_N_2_ complexes, PA@C_5_N_2_ shows larger orbitals density isosurface, depicting the major shift in electronic density between C_5_N_2_ and picric acid. This visual representation is analogous to the reduced HOMO–LUMO energy gap resulting in excellent sensitivity of picric acid toward the C_5_N_2_ as compared to the rest of the analytes.

**Fig. 6:**
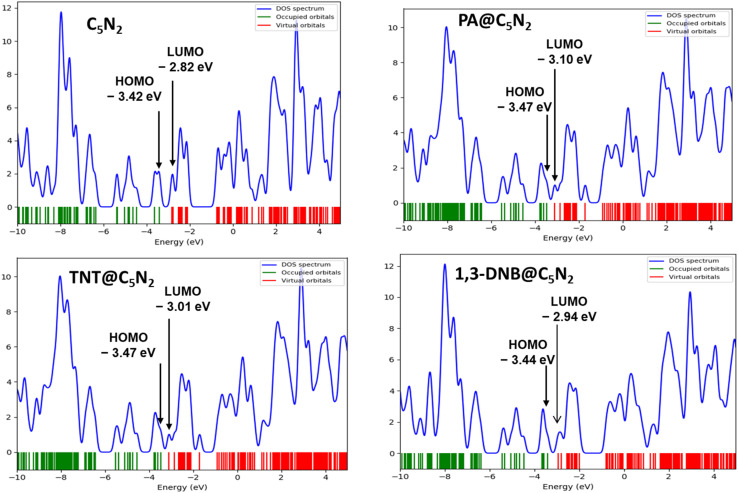
DOS spectra of the pristine C_5_N_2_, PA@C_5_N_2_, TNT@C_5_N_2_ and 1,3-DNB@C_5_N_2_.

Electronic properties of considered complexes are further executed by the DOS analysis. The spectra of DOS are plotted in Fig. 6 for a better understanding of interactions after adsorptions of analytes. Density of state spectra reveals that after adsorption of analytes, virtual orbitals are shifted more negatively, resulting in a reduced energy gap. In the DOS spectra, it is observed that HOMO appeared at −3.47 eV for PA@C_5_N_2_ and TNT@C_5_N_2_ as compared to pristine C_5_N_2_ sheet (−3.42 eV) while the LUMO virtual orbitals are shifted to −3.10 eV and −3.01 eV from −2.82 eV of C_5_N_2_, respectively. The formation of new orbitals of HOMO and LUMO results in a reduction of energy gap as compared to pristine substrate. A similar pattern is also observed for the 1,3-DNB@C_5_N_2_ complex. Among the studied systems, the more pronounced shift is observed for the PA@C_5_N_2_ system, thus lowering the energy gap. The shifting of orbitals and their peak intensities reflect the conductivity and sensitivity of C_5_N_2_ towards analytes, validating FMO results.^76^

### 3.4 NBO and EDD analysis

Natural bond orbital (NBO) analysis provides a deeper evaluation of the interaction mechanism by determining the amount of charge transfer between the nitroaromatic analytes and C_5_N_2_ substrate upon complexation. The NBO results of nitroaromatics@C_5_N_2_ complexes are presented in Table 3. The values of NBO charges are negative on the 1,3-DNB, TNT and PA in nitroaromatics@C_5_N_2_ complexes (presented in Table 3), which shows that charges are transferred from the sheet towards the nitroaromatics analytes. For the TNT@C_5_N_2_ complex, 0.406 *e* charge is shifted from the C_5_N_2_ substrate to TNT. Similarly, 0.426 *e* charge is being transferred from substrate to PA in case of PA@C_5_N_2_ complex while for 1,3-DNB@C_5_N_2_ complex, the amount of charge transferred from substrate to the analyte is only 0.208 *e*. From these results, it is deduced that PA extracted the highest amount of charge from the C_5_N_2_ substrate as compared to TNT and 1,3-DNB.^78^

**Fig. 7:**
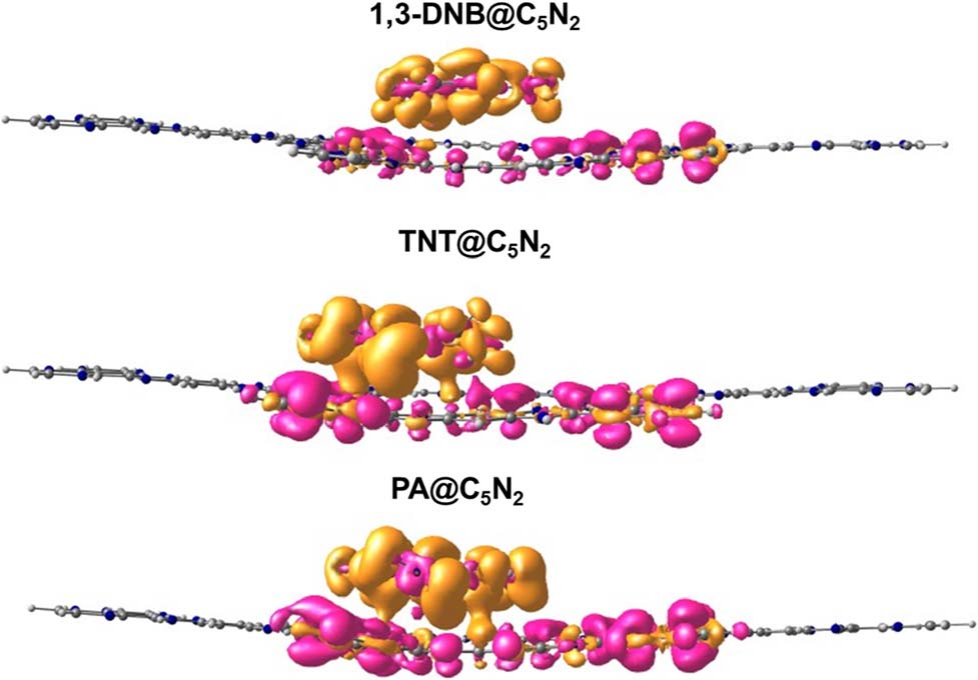
EDD plots of nitroaromatics@C_5_N_2_ complexes. The orange color isosurface shows the charge-accumulated region and the pink color isosurface represents the charge-depleted region.

Electron density difference (EDD) analysis helps to visualize the electronic density distribution among the nitroaromatics analytes and substrate upon complexation. The difference in the electronic density of the nitroaromatics@C_5_N_2_ complex and the sum of the electronic density of the nitroaromatic analytes and C_5_N_2_ substrate, taken separately, gives the EDD results.^44^ The EDD plots are given in the Fig. 7. In EDD plots, the transfer of electronic density is depicted by the orange (electronic density accumulated region) and pink (electronic density depleted region) colored isosurface, at the interacting sites of nitroaromatics and C_5_N_2_ substrate. EDD results of 1,3-DNB@C_5_N_2_ and TNT@C_5_N_2_ show that nitroaromatics are mainly covered with orange isosurface, indicating the analytes withdraw electron density from C_5_N_2_ substrate. In the EDD plot of picric acid@C_5_N_2_ complex, the analyte is covered primarily with orange isosurface which depicts the shifting of electron density from pyrazine and benzene ring of C_5_N_2_, making them depleted region shown by pink color isosurface. The orange isosurface covering the phenolic group of picric acid along with three nitro groups signifies strong extraction of electrons from the benzene ring of C_5_N_2_ substrate as compared to TNT@C_5_N_2_ and 1,3-DNB@C_5_N_2._ These results show consistency with NBO analysis.

### 3.5 Recovery time

Recovery time is one of the parameters to determine the reproducibility of C_5_N_2_ material as a sensor. Generally, thermal effects are applied to calculate the recovery time through transition state theory.^79^ The equation for calculating recovery time is as follows:

5 *τ* = *ν*^−1^e^−*E*_ads_/*KT*^

The *τ* stands for the recovery time, *ν* represents the attempt frequency (10^14^ s^−1^),^45,80,81^*E*_ads_ express the adsorption energy, *K* denotes Boltzmann constant (1.99 × 10^−3^ kcal mol^−1^ K^−1^), while *T* represents the temperature. For calculating the recovery time for the nitroaromatics@C_5_N_2_, different temperatures are employed. Generally, recovery time is directly proportional to interaction energies. Therefore, the recovery times for 1,3-DNB@C_5_N_2_, TNT@C_5_N_2_ and PA@C_5_N_2_ are, 5.24 × 10^−4^ s, 4.10 s and 74.74 s, respectively at 473 K. The recovery time of the nitroaromatics@C_5_N_2_ at different temperatures is presented in the Table S3.† It is observed that the systems' recovery time decreases with an increase in temperature.^81^

## 4 Conclusion

DFT calculations have been employed to determine the sensing capability of C_5_N_2_ substrate towards the lethal nitroaromatic compounds including 1,3-dinitrobenzene, trinitrotoluene and picric acid. Structures of nitroaromatics, C_5_N_2_ sheet, and their complexes have been optimised at the PBE0-D3BJ/def2SVP level of theory. Nitroaromatics are placed at five positions with different orientations to get the most stable geometry. Among studied systems, PA@C_5_N_2_ is observed with high interaction energy of −34.37 kcal mol^−1^. The trend in interaction is as follows: PA@C_5_N_2_ > TNT@C_5_N_2_ > 1,3-DNB@C_5_N_2_. Geometrical and electronic properties are determined for better understanding of the nature and type of interactions. QTAIM and NCI analyses confirm the existence of non-covalent interactions. The presence of denser green isosurface in 3D, along with bluish-green spots in the 2D RDG graph, shows the strong non-covalent interactions in the PA@C_5_N_2_ complex. These NCI results are verified by small and positive values of Laplacian and electronic density obtained through topological parameters. The conductivity of C_5_N_2_ has been increased after the adsorption of analytes due to a reduction in the HOMO–LUMO gap. More significant change is observed for PA@C_5_N_2_ (0.37) which shows selectivity of C_5_N_2_ towards the PA. FMO results are also confirmed by DOS analysis, which shows the prominent shifting of virtual orbital after the adsorption of PA. Charge transfer (NBO) analysis also shows that among the studied systems, significant charge transfer is observed for the PA@C_5_N_2_ system. Furthermore, EDD analysis confirms the NBO analysis by providing visual illustrations. Recovery time for all the studied complexes has been computed using the transition state theory equation. The results of recovery time follow the interaction energies; however, recovery time could be appreciably reduced by increasing temperature. These results show that the C_5_N_2_ substrate could be an efficient electrochemical sensor towards toxic nitroaromatics.

## Data availability

The authors confirm that the data supporting the findings of this study are available within the article [and/or] its ESI.† Any further information will also be available upon request from the authors

## Conflicts of interest

There are no conflicts of interest to declare.

## Supplementary Material

RA-014-D4RA05600K-s001
