# Supplementary material for: Detection of nitro-aromatics using C5N2 as an electrochemical sensor: a DFT approach
Source: RSC Adv. 2024 Sep 23;14(41):30116–26. doi: 10.1039/d4ra05600k (PMC11417677; doi:10.1039/d4ra05600k)
Supplement: RA-014-D4RA05600K-s001 [file RA-014-D4RA05600K-s001.pdf]

Supplementary Information (SI) for RSC Advances.
This journal is © The Royal Society of Chemistry 2024

Supplementary Information

for

Detection of Nitro-Aromatics using C_5N_2 as an Electrochemical Sensor: a DFT approach

Nabeela[1], Muhammad Ali Hashmi[1,2*], Ahmad Nauman Shah Saqib[1], Aqsa Kamran[1], Ahmed Lakhani[3,**]

[1] *Department of Chemistry, Division of Science & Technology, University of Education, 54770 Lahore, Pakistan*

[2] *School of Chemical and Physical Sciences, Victoria University of Wellington, Wellington 6012, New Zealand*

[3] *Department of Biomedical and Health Sciences, Calumet College of St. Joseph, 2400, New York Ave, Whiting, IN, 46394, USA*

***** Correspondence: muhammad.hashmi@ue.edu.pk (MAH); alakhani@ccsj.edu (AL)

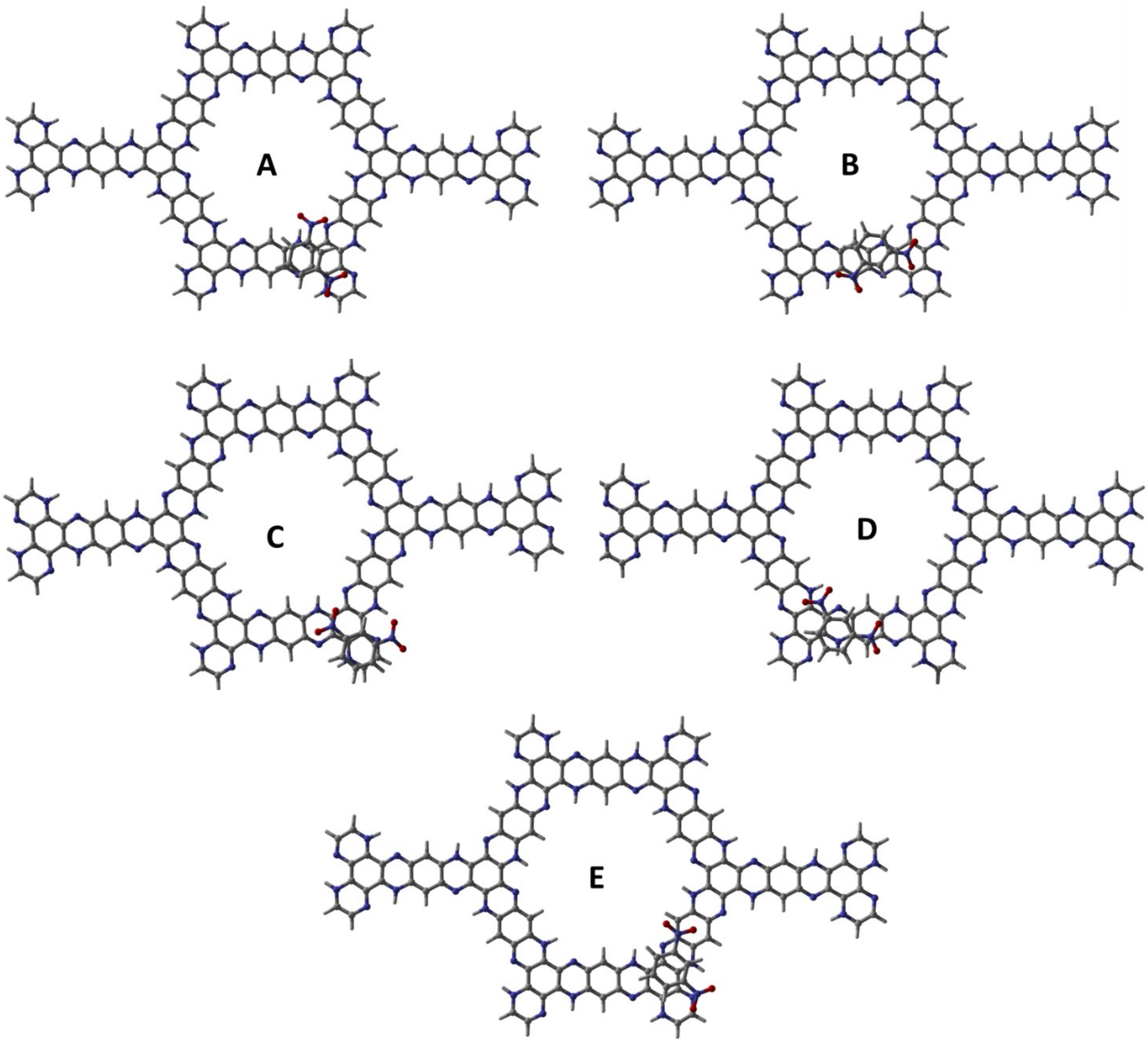

Fig. S1 : Optimized geometry of 1,3-DNB@C_5N_2 with all positions. The positions are; central cavity (A), triazine site (B), benzene ring (C), hydrogenated benzene ring (D) and pyrazine ring (E).

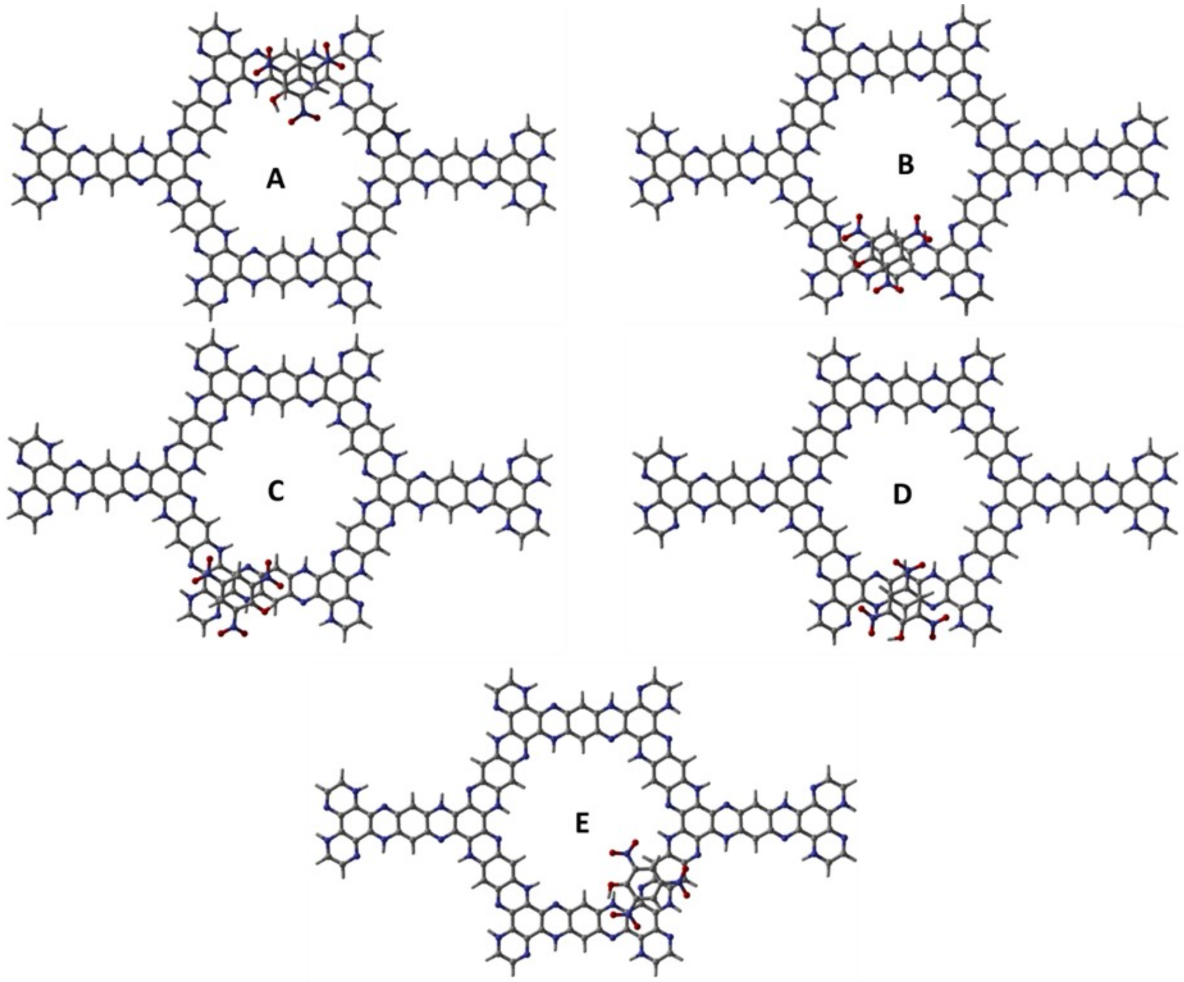

Fig. S2: Optimized geometries of PA@C_5N_2 with all possible positions. The positions are; central cavity (A), triazine site (B), benzene ring (C), hydrogenated benzene ring (D) and pyrazine ring (E).

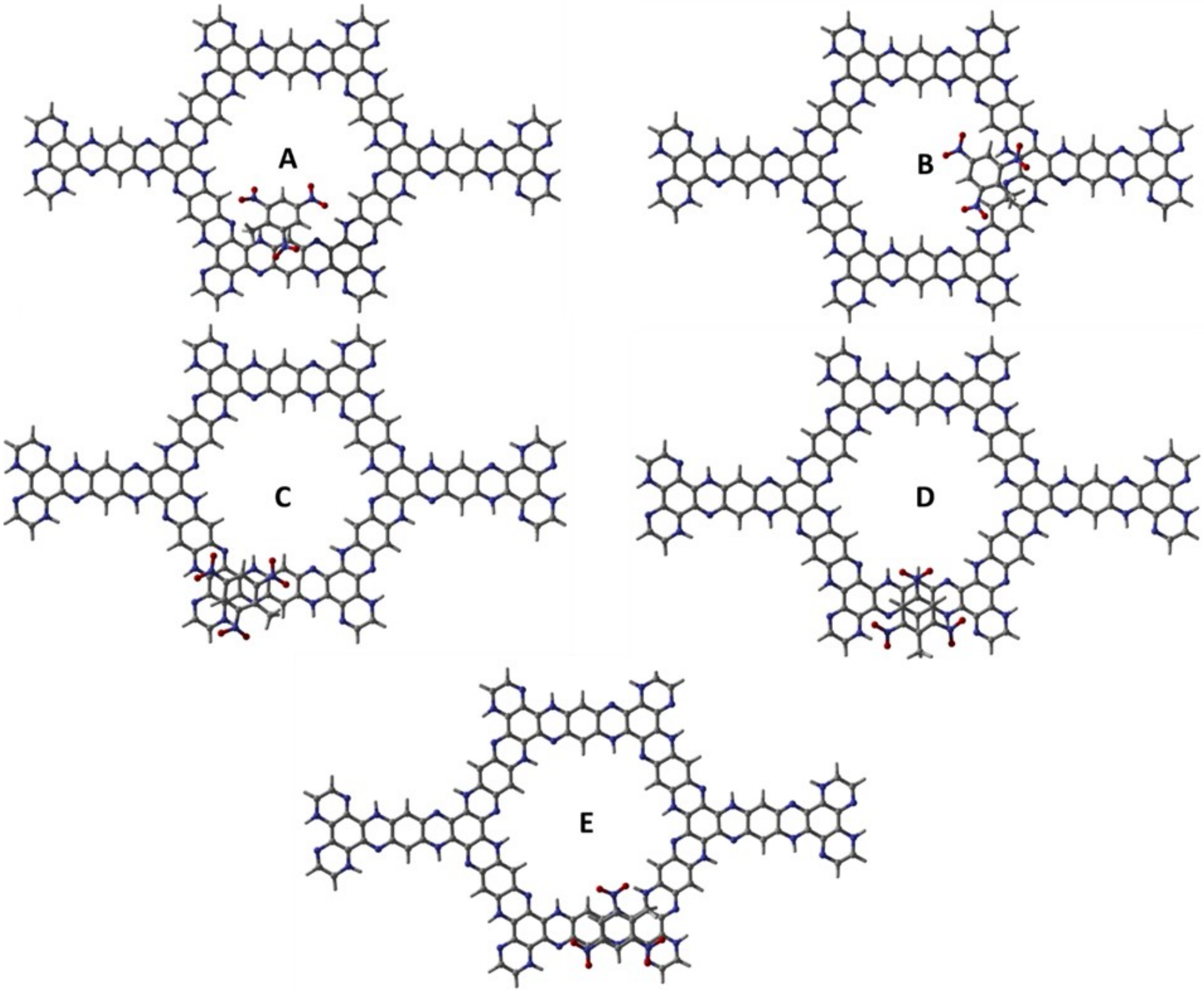

Fig. S3: Optimized geometries of TNT@C_5N_2 with all possible positions. The positions are; central cavity (A), triazine site (B), benzene ring (C), hydrogenated benzene ring (D) and pyrazine ring (E).

Table S1 Interaction energies of all analytes@C_5N_2 Complexes at possible positions. The positions are; central cavity (A), triazine site (B), benzene ring (C), hydrogenated benzene ring (D) and pyrazine ring (E).

Analyte at specific position of C_5N_2	Interaction Energies (Kcal/mol)	Analyte at specific position of C_5N_2	Interaction Energies (Kcal/mol)	Analyte at specific position of C_5N_2	Interaction Energies (Kcal/mol)
TNT@C_5N_2-A	-25.01	PA@C_5N_2-A	-30.41	1-3-DNB@C_5N_2-A	-22.80
TNT@C_5N_2-E	-30.36	PA@C_5N_2-E	-30.38	1-3-DNB@C_5N_2-E	-21.82
TNT@C_5N_2-B	-30.92	PA@C_5N_2-B	-29.23	1-3-DNB@C_5N_2- B	-20.48
**TNT@C_5N_2-C**	**-31.64**	**PA@C_5N_2-C**	**-34.37**	1-3-DNB@C_5N_2-C	-22.38
TNT@C_5N_2-D	-30.35	PA@C_5N_2-D	-33.70	**1-3-DNB@C_5N_2-D**	**-23.21**

Table S2 QTAIM parameter for 1,3-DNB@C_5N_2 and TNT@C_5N_2

**Analyte--C_5N_2**	**$\nabla_2\rho$**	**ρ**	**V(r)**	**H**	**G(r)**
1,3-DNB@C_5N_2					
O 13 -- C 1	0.030	0.009	-0.006	0.0009	0.007
C 12 -- C6	0.030	0.010	-0.005	0.0012	0.006
C 8 -- C3	0.025	0.009	-0.004	0.0010	0.005
O11 -- N7	0.033	0.009	-0.007	0.0006	0.008
C 14 -- N4	0.019	0.008	-0.004	0.0004	0.004
C10 -- C5	0.027	0.011	-0.005	0.0008	0.006
C 9 -- C 2	0.025	0.008	-0.004	0.0011	0.005
TNT@C_5N_2					
O5 -- C10	0.028	0.008	-0.005	0.0009	0.006
O2 -- N6	0.025	0.007	-0.005	0.0008	0.005
N13 -- N11	0.037	0.010	-0.006	0.0014	0.008
N16 -- C18	0.028	0.009	-0.005	0.0010	0.006
H19 -- C21	0.028	0.010	-0.005	0.0007	0.006
O 17 -- C20	0.026	0.008	-0.005	0.0009	0.005
C15 -- N22	0.023	0.008	-0.004	0.0007	0.005
C14 -- C12	0.027	0.009	-0.004	0.0012	0.006
C4 -- C9	0.035	0.014	-0.007	0.0010	0.008
N3 -- C7	0.030	0.009	-0.005	0.0011	0.006
O1 -- N8	0.028	0.009	-0.006	0.0005	0.006

Table.S3 Recovery time of nitroaromatics at different temperatures (K)

Analyte-Complex Systems	Recovery Time (298K)	Recovery Time (398K)	Recovery Time (473K)
PA@C_5N_2	1.57×10^{11} s	7.33×10^{4}s	74.74 s
TNT@C_5N_2	1.56×10^{9} s	2.32 ×103s	4.10 s
1,3-DNB@C_5N_2	1.03×10^{3}s	1.13×10^{-14} s	5.24×10^{-4} s
